# *Notes from the Field:* Fatal Pneumonic Plague — Coconino County, Arizona, July 2025

**DOI:** 10.15585/mmwr.mm7538a2

**Published:** 2026-10-01

**Authors:** Christine A. Wang, Jacob Hojnacki, Sierra Jaramillo, Saba Qasmieh, Irene Ruberto, Laura Platt, Schante Patton, Guillermo Adame, Drew Francis, Grace Marx, Shane Brady, Kenneth K. Komatsu, Kathryn Burr, Joel Terriquez

**Affiliations:** ^1^Epidemic Intelligence Service, CDC; ^2^Arizona Department of Health Services; ^3^Coconino Department of Health and Human Services, Flagstaff, Arizona; ^4^Division of Vector-borne Diseases, National Center for Emerging and Zoonotic Infectious Diseases, CDC; ^5^Career Epidemiology Field Officer Program, CDC.

**Summary**

**What is already known about this topic?**

Pneumonic plague is a rare and often fatal disease that can be transmitted by respiratory droplets from animals or persons infected with *Yersinia pestis* bacteria.

**What is added by this report?**

In July 2025, Arizona reported its first pneumonic plague death since 2007, in a man with occupational exposure to ill cats. Two days after the patient’s death, preliminary diagnostic test results supported a suspected pneumonic plague diagnosis, prompting immediate identification of potentially exposed persons for postexposure prophylaxis.

**What are the implications for public health practice?**

In areas with endemic plague, preliminary diagnostic test results combined with clinical suspicion for pneumonic plague can result in timely public health interventions.

Pneumonic plague is transmitted through inhalation of respiratory droplets containing the bacterium *Yersinia pestis* and has a nearly 100% case-fatality rate when untreated (*1*). Although *Y. pestis* is endemic among rodents in northern Arizona, only eight human plague cases were reported in Arizona during 2000–2024. In July 2025, Arizona reported its first pneumonic plague death since 2007 (*2*).

## Investigation and Outcomes

In July, a young adult male was evaluated in a Coconino County, Arizona emergency department after 2 days of abdominal pain, chest pain, and dry cough (Figure). He was afebrile, tachycardic, and tachypneic, and had leukocytosis (28.3 ×10^3 ^white blood cells/*µ*L). Chest radiographs and computed tomographic angiography of the chest, abdomen, and pelvis identified pulmonary infiltrates consistent with pneumonia; no buboes (i.e., painful, swollen lymph nodes) were observed. Clinicians initially suspected hantavirus disease or bacterial pneumonia. The patient was admitted and received empiric antibiotic treatment with piperacillin-tazobactam, vancomycin, and azithromycin. Despite progressively intensive oxygen therapy, his hypoxemia worsened, and he died <8 hours after admission.

**FIGURE:**
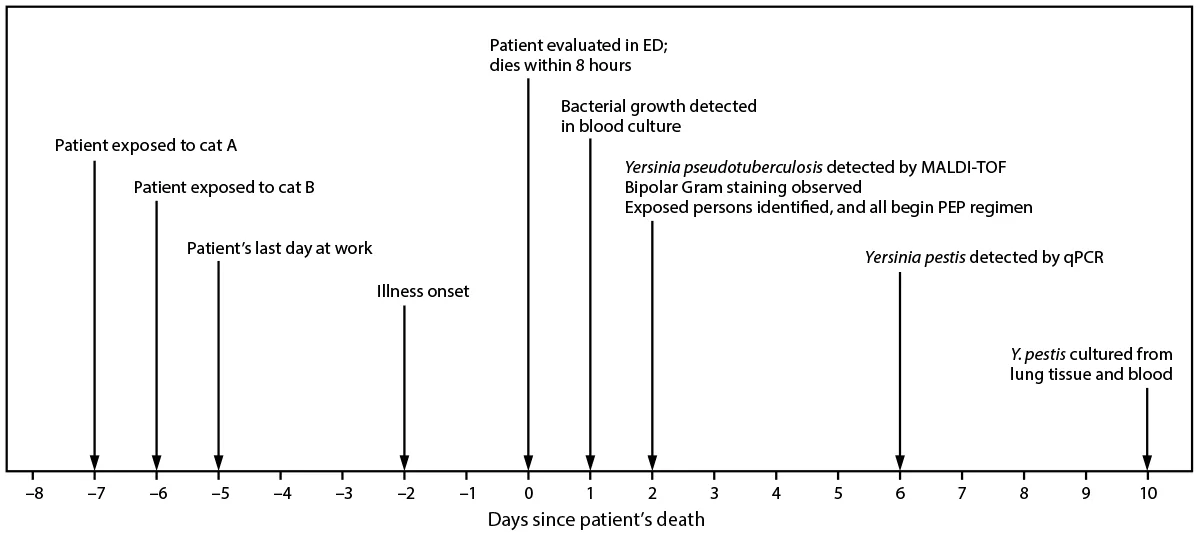
Timeline of events associated with a fatal case of pneumonic plague — Coconino County, Arizona, July 2025 **Abbreviations**: ED = emergency department; MALDI-TOF = matrix-assisted laser desorption/ionization time-of-flight; PEP = postexposure prophylaxis; qPCR = quantitative polymerase chain reaction.

One day later, bacterial growth was detected in the patient’s blood cultures. The following day, matrix-assisted laser desorption/ionization time-of-flight (MALDI-TOF) mass spectrometry of bacterial growth from that blood culture identified *Yersinia. pseudotuberculosis,* and bipolar-staining gram-negative bacilli were identified (Supplementary Figure). Together, both results raised suspicion for pneumonic plague, and the local public health department was immediately notified.

Two days after the patient’s death, 46 potentially exposed persons were identified, and all commenced a 7-day course of oral ciprofloxacin postexposure prophylaxis (PEP) (*3*). Potentially exposed persons included household members, coworkers, and hospital staff members; no contact became ill. Six days after the patient’s death, the Arizona State Public Health Laboratory (ASPHL) detected *Y. pestis* from initial blood cultures using quantitative (real-time) polymerase chain reaction testing. Four days later (10 days after the patient’s death), ASPHL isolated *Y. pestis* from blood and lung tissue cultures.

The public health investigation focused on patient exposures during the pneumonic plague incubation period of 1–5 days preceding illness onset. Family members reported that the patient did not engage in outdoor activities and only spent time at his workplace or residence. Five and 4 days before his illness onset, he had occupational exposure to two indoor domestic cats with respiratory symptoms and other signs of systemic illness consistent with pneumonic plague. Inhalation transmission from either cat was possible with the patient’s occupational exposure. Feline specimens were unavailable for *Y. pestis* testing because both cats had been euthanized before the patient’s illness onset. No other persons exposed to these cats reported illness.

CDC was consulted after the patient’s death and primarily focused on supporting the interpretation of clinical signs for each animal to which the patient was exposed. This activity was reviewed by CDC, deemed not research, and conducted consistent with applicable federal law and CDC policy.*

## Preliminary Conclusions and Actions

*Y. pestis* misidentification can occur with automated pathogen detection systems, including MALDI-TOF (*4*). The combination of Gram stain test results, MALDI-TOF results, the patient’s clinical signs and symptoms, and known endemicity of *Y. pestis* in northern Arizona sufficiently raised suspicion for pneumonic plague. These circumstances supported prompt identification of persons potentially exposed to the patient for PEP initiation 8 days before confirmation of *Y. pestis*.

Health care providers in areas where plague is endemic should consider plague in patients with severe respiratory and systemic disease who have been exposed to ill animals. Appropriate laboratory safety practices must also be considered for clinical laboratory personnel handling specimens from these patients. Pneumonic plague risk increases with occupational exposure to animals infected with *Y. pestis*, especially cats, because of their susceptibility to plague (*5*). During evaluation of ill animals and persons suspected of having plague or handling their clinical specimens, consistent use of personal protective equipment, especially appropriate respiratory protection, can reduce occupational exposures to zoonotic pathogens such as plague among veterinary, medical, and clinical microbiology personnel.

## Footnotes

* 45 C.F.R. part 46.102(l)(2), 21 C.F.R. part 56; 42 U.S.C. Sect. 241(d); 5 U.S.C. Sect. 552a; 44 U.S.C. Sect. 3501 et seq.
